# Understanding Cognitive Deficits in People with High Blood Pressure

**DOI:** 10.3390/jpm13111592

**Published:** 2023-11-10

**Authors:** Weixi Kang, Sònia Pineda Hernández

**Affiliations:** 1 Department of Brain Sciences, Imperial College London, London W12 0NN, UK; 2 School of Health Sciences, University Pompeu Fabra, 08002 Barcelona, Spain

**Keywords:** high blood pressures, dementia, cognitive deficits, cognitive performance

## Abstract

High blood pressure is associated with an elevated risk of dementia. However, much less is known about how high blood pressure is related to cognitive deficits in domains including episodic memory, semantic verbal fluency, fluid reasoning, and numerical ability. By analyzing data from 337 participants (57.39% female) with a history of clinical high blood pressure diagnosis with a mean age of 48.78 ± 17.06 years and 26,707 healthy controls (58.75% female) with a mean age of 45.30 ± 15.92 years using a predictive normative modeling approach and one-sample t-tests, the current study found that people with high blood pressure have impaired immediate (t(259) = −4.71, *p* < 0.01, Cohen’s d = −0.08, 95% C.I. [−0.11, −0.05]) and delayed word recall (t(259) = −7.21, *p* < 0.01, Cohen’s d = −0.11, 95% C.I. [−0.15, −0.08]) performance. Moreover, people with high blood pressure also exhibited impaired performance in the animal naming task (t(259) = −6.61, *p* < 0.0001, Cohen’s d = −0.11, 95% C.I. [−0.15, −0.08]), and number series (t(259) = −4.76, *p* < 0.01, Cohen’s d = −0.08, 95% C.I. [−0.11, −0.05]) and numeracy tasks (t(259) = −4.16, *p* < 0.01, Cohen’s d = −0.06, 95% C.I. [−0.09, −0.03]) after controlling for demographic characteristics. Clinicians and health professionals should consider including these tasks as part of the neuropsychological assessment for people with high blood pressure, to detect their cognitive deficits. Moreover, they should also come up with ways to improve cognitive performance in people with high blood pressure.

## 1. Introduction

High blood pressure is a health condition identified by increased levels of blood pressure, which is a prevalent health issue worldwide. Cognition refers to the mental processes and abilities involved in acquiring knowledge, understanding, reasoning, and memory. Studies have shown a significant link between elevated blood pressure and cognitive decline, with high blood pressure being connected to a higher likelihood of experiencing cognitive impairment, vascular dementia, and Alzheimer’s disease [1,2,3,4,5]. Understanding cognition in the context of high blood pressure is crucial for several reasons. Firstly, a decline in cognitive function can have a substantial impact on an individual’s quality of life and their capacity to perform routine activities. Secondly, damage to the brain blood vessels caused by elevated blood pressure can play a role in the progression of cognitive decline and the onset of dementia [6]. Moreover, treating high blood pressure and managing other cardiovascular risk factors have been shown to slow down cognitive decline and reduce the risk of dementia. Therefore, recognizing and addressing cognitive changes in individuals with high blood pressure can help in the early detection, appropriate management, and implementation of strategies to preserve cognitive function, ultimately improving the overall health outcomes [7].

The identification and management of high blood pressure are regarded as crucial objectives in terms of reducing the global burden of dementia [8]. While there have been global improvements in detecting high blood pressure, the levels of treatment and control vary. In 2019, a study found that the control rates were 23% for women and 18% for men. These rates were even lower in low- to middle-income countries, where the prevalence of high blood pressure is increasing. Disparities in access to medications, the absence of universal healthcare, and limited public health measures may explain these low control rates, ultimately leading to an increased burden of conditions related to high blood pressure, such as heart disease, chronic kidney disease (CKD), and dementia [9].

The majority of prospective cohort studies suggest a positive association between elevated blood pressure and the likelihood of experiencing cognitive impairment and dementia [1,2,3,4,5]. The strongest connection is seen between high blood pressure in midlife and the subsequent risk of cognitive decline and new-onset dementia. In a recent meta-analysis of observational studies, which compiled information from 135 prospective cohort studies (including three with nested designs) covering more than 2 million individuals, a significant link was established between prior history of high blood pressure in middle age (risk ratio 1.20; 95% confidence interval 1.06–1.35), increased systolic blood pressure (risk ratio 1.54; 95% confidence interval 1.25–1.89) and diastolic blood pressure (risk ratio 1.50; 95% confidence interval 1.04–2.16), and the likelihood of developing dementia. This analysis found a higher risk is associated with systolic blood pressure levels above 130 mmHg [6]. In contrast, among older individuals, there was no general connection between high blood pressure and the risk of dementia. However, a notable association was detected during the transition from mild cognitive impairment to dementia (risk ratio 1.41; 1.00–1.99). In contrast to blood pressure levels in midlife, the risk of dementia was linked to systolic blood pressure exceeding 180 mmHg in older age groups (risk ratio 1.45; 95% confidence interval 1.03–2.06). Interestingly, among older age groups, there seemed to be a potential protective influence of diastolic blood pressure against dementia risk (risk ratio 0.77; 95% confidence interval 0.59–1.00 for the diastolic blood pressure of 90 mmHg or higher). This effect might be attributed to the emergence of competing blood pressure mechanisms such as orthostatic hypotension. Importantly, these analyses and others pointed to varying risks based on ethnicity, with higher risks observed among older Black populations compared to other ethnic groups [10].

While previous research has examined the connections between high blood pressure and the likelihood of dementia, there is relatively limited knowledge regarding how high blood pressure correlates with deficits in various cognitive areas, such as episodic memory, semantic verbal fluency, fluid reasoning, and numerical aptitude.

## 2. Methods

### 2.1. Data

The Understanding Society: UK Household Longitudinal Study (UKHLS) data were utilized in our study. Since 1991, data have been collected yearly from the original sample of UK households [11]. During Wave 1, all the participants were asked about whether they had received a formal diagnosis of high blood pressure, which occurred between 2009 and 2010. Subsequently, in each wave up to Wave 3, individuals were once more questioned if they had recently received a diagnosis of high blood pressure. In addition, during Wave 3, participants completed psychological and demographic distress questionnaires (collected between 2011 and 2012). Thus, there were 337 participants (57.39% female) with a history of clinical high blood pressure diagnosis with a mean age of 48.78 ± 17.06 years and 26,707 healthy controls (58.75% female) with a mean age of 45.30 ± 15.92 years.

### 2.2. Measures

#### 2.2.1. High Blood Pressure

Self-reported high blood pressure is a reliable indicator for identifying high blood pressure in the general population [12,13]. The question “Has a doctor or other health professional ever told you that you have any of these conditions? High blood pressure.” was used to detect high blood pressure during Wave 1. In the subsequent waves, participants were asked about whether they had received a recent clinical diagnosis of high blood pressure.

#### 2.2.2. Cognitive Abilities

Episodic memory was evaluated through tasks involving immediate and delayed word recall. Semantic verbal fluency was assessed using an animal fluency task [14,15,16]. Fluid reasoning, which involves the ability to solve novel problems using abstract concepts, was measured using a number series assignment, akin to logic problems [14]. The numerical ability tests encompassed problem-solving scenarios encountered in daily life. An example question from this category is: “Before a sale, a sofa costs £300. If all items are sold at half price during the sale, what will be the cost of the sofa?” For more detailed information on the procedures for these tasks, refer to the following link: https://www.understandingsociety.ac.uk/sites/default/files/downloads/documentation/mainstage/user-guides/6614_Cognitive_Ability_measures_v1.1.pdf (accessed on 10 July 2023).

#### 2.2.3. Demographic Controls

In the model, we included demographic variables such as age (as a continuous variable), gender (coded as 1 for male and 2 for female), educational attainment (coded as 1 for college and 2 for below college), marital status (coded as 1 for single and 2 for married), monthly income (as a continuous variable), and place of residence (coded as 1 for urban and 2 for rural) as the control factors.

### 2.3. Statistical Analyses

A predictive normative modeling approach was used to analyze the current dataset. All the cognitive measure scores were standardized before further analyses. First, five generalized linear models were constructed by taking demographic variables, including age, sex, highest educational qualification, legal marital status, monthly income, and residence, as the predictors of cognitive measures, including immediate and delayed word recall, animal naming, numerical ability, and the number series task, as the predicted variables. Second, demographic controls from patients with high blood pressure were added into these generalized linear models as inputs to predict the expected scores of these cognitive tasks given their demographics. Finally, the expected scores were subtracted from the actual scores, and one-sample t-tests were used to determine if these differences significantly differed from zero. This method has more benefits than paired-sample t-tests since it can account for demographic factors that may influence these cognitive skills, as well as cope with an unequal sample size.

## 3. Results

The descriptive statistics can be found in Table 1. Here, we first report the results from the model trained using the healthy controls. There was a main effect of age (F(1, 5109) = 436.34, *p* < 0.001), sex (F(1, 5109) = 97.18, *p* < 0.001), monthly income (F(1, 5109) = 15.70, *p* < 0.001), highest educational qualification (F(1, 5109) = 185.44, *p* < 0.001), marital status (F(1, 5109) = 8.96, *p* < 0.01), and residence (F(1, 5109) = 10.26, *p* < 0.01) on the immediate word recall task. There was also a significant main effect of age (F(1, 5109) = 437.14, *p* < 0.001), sex (F(1, 147.24) = 96.54, *p* < 0.001), monthly income (F(1, 5109) = 7.36, *p* < 0.01), highest educational qualification (F(1, 5109) = 132.54, *p* < 0.001), marital status (F(1, 5109) = 7.25, *p* < 0.01), and residence (F(1, 5109) = 11.44, *p* < 0.001) on the delayed word recall task. There was also a significant main effect of age (F(1, 5109) = 289.01, *p* < 0.001), sex (F(1, 5109) = 8.36, *p* < 0.01), monthly income (F(1, 5109) = 12.69, *p* < 0.001), highest educational qualification (F(1, 5109) = 107.85, *p* < 0.001), marital status (F(1, 5109) = 12.41, *p* < 0.001), and residence (F(1, 5109) = 25.15, *p* < 0.001) on the animal naming task. There was a significant main effect of age (F(1, 5109) = 121.35, *p* < 0.001), sex (F(1, 5109) = 51.68, *p* < 0.001), monthly income (F(1, 5109) = 44.96, *p* < 0.001), highest educational qualification (F(1, 5109) = 287.22, *p* < 0.001), marital status (F(1, 5109) = 39.52, *p* < 0.001), and residence (F(1, 5109) = 16.03, *p* < 0.001) on the number series task. Finally, there was a significant effect of age (F(1, 5109) = 23.68, *p* < 0.001), sex (F(1, 5109) = 185.65, *p* < 0.001), monthly income (F(1, 5109) = 44.27, *p* < 0.001), highest educational qualification (F(1, 5109) = 286.15, *p* < 0.001), marital status (F(1, 5109) = 45.16, *p* < 0.001), and residence (F(1, 5109) = 26.55, *p* < 0.001) on numeracy (Table 2).

**Figure 1:**
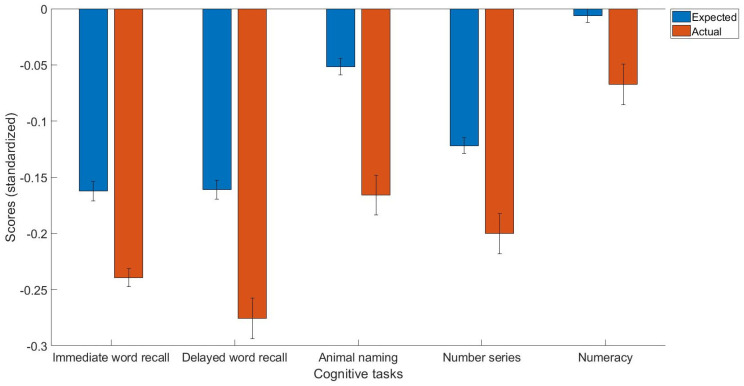
The expected and actual scores for the cognitive measures, including immediate word recall, delayed word recall, animal naming, number series, and numeracy tasks in people with high blood pressure. All the scores are standardized.

The main findings were that people with high blood pressure had impaired immediate (t(259) = −4.71, *p* < 0.01, Cohen’s d = −0.08, 95% C.I. [−0.11, −0.05]) and delayed word recall (t(259) = −7.21, *p* < 0.01, Cohen’s d = −0.11, 95% C.I. [−0.15, −0.08]) performance. Moreover, people with high blood pressure also exhibited impaired performance in the animal naming task (t(259) = −6.61, *p* < 0.0001, Cohen’s d = −0.11, 95% C.I. [−0.15, −0.08]), and the number series (t(259) = −4.76, *p* < 0.01, Cohen’s d = −0.08, 95% C.I. [−0.11, −0.05]) and numeracy tasks (t(259) = −4.16, *p* < 0.01, Cohen’s d = −0.06, 95% C.I. [−0.09, −0.03]; Figure 1).

## 4. Discussion

The current study aimed to explore cognitive deficits in domains including episodic memory, semantic verbal fluency, fluid reasoning, and numerical ability in people with high blood pressure. The results revealed that people with high blood pressure exhibit impaired performance in all cognitive domains compared to people without high blood pressure. These results provide novel findings regarding how high blood pressure is negatively related to episodic memory, semantic verbal fluency, fluid reasoning, and numerical ability, broadly consistent with studies that have reported a positive link between high blood pressure and the risk of cognitive impairment and dementia [1,2,3,4,5].

There are various potential pathways that may clarify the link between high blood pressure and a decline in cognitive function. For instance, elevated blood pressure is a major contributing factor to the onset of CKD. Within the CKD population, cognitive impairment is prevalent, ranging from 10% to 40% [17,18]. Approximately 50% of people receiving hemodialysis experience significant cognitive declines, ranging from moderate to severe [19]. Both the reduced estimated glomerular filtration rate and presence of albuminuria independently raise the risk of cognitive impairment and dementia [20]. The connection between albuminuria and cognitive impairment primarily stems from a shared mechanism involving damage to vascular endothelial function. Chronic uremia in CKD can disturb the blood–brain barrier’s integrity, which can lead to the development of cerebral small-vessel ischemia [21]. In individuals undergoing dialysis, there are other factors that can also play a role, such as variations in blood pressure during ultrafiltration, an insufficient autonomic response to these changes, and the occurrence of cerebral stunning, all of which can result in cerebral damage and decreased blood circulation [22,23]. Other mechanisms through which CKD contributes to cognitive impairment encompass vascular calcification and arteriosclerosis [24]. CKD represents a standalone risk factor for both sudden and hidden strokes, distinct from hypertension, and has been linked to elevated beta-amyloid generation and hindered beta-amyloid removal [25].

Some research indicates the common progression of diseases [26], where elevated blood pressure concurrently contributes to both chronic kidney disease and cerebrovascular disease. Both the kidneys (specifically, the afferent arterioles) and the brain (particularly the deep perforating arterioles) are exposed to increased pressure levels, rendering them susceptible to damage from hypertension and issues with autoregulation, as proposed in the strain vessel hypothesis [27]. There is a prolonged period during which damage from high blood pressure to the kidneys can occur before a decline in kidney function, which is analogous to the impact of sustained high blood pressure on cognitive function and may be accelerated by other cardiovascular events [28]. In randomized controlled trials concentrating on blood pressure reduction, the relative risk reduction in kidney-related outcomes corresponds to estimates of cognitive outcomes, as suggested by indirect comparisons of the meta-analyses and data from the SPRINT trial [29,30].

The presence of large vessel atherosclerosis is linked to a heightened likelihood of experiencing ischemic strokes and an elevated risk of developing Alzheimer’s disease [31]. Furthermore, high blood pressure contributes to the age-related stiffening of elastic arteries in the aortic arch and major vessels, which play a vital role in buffering and dampening hemodynamic fluctuations, known as the Windkessel effect. Elevated blood pressure leads to increased pulsatile pressure within the brain. In older individuals with high blood pressure, this heightened pulsatile pressure places greater stress on the cerebral microcirculation [32].

Elevated blood pressure significantly increases the risk of heart failure (risk ratio 1.40; 95% confidence interval 1.24–1.59), contributing to approximately 10.1% of cases, as determined by the analysis of the NHANES dataset [33]. Heart failure, on its own, is an independent risk factor for dementia, elevating the odds by 28%. Additionally, high blood pressure is a major contributor to atrial fibrillation, which is in turn associated with an elevated risk of cognitive impairment [34]. This link is primarily attributed to the increased risk of thromboembolism. According to this meta-analysis involving 43 cohort studies [34], atrial fibrillation is correlated with 50% higher relative odds of experiencing cognitive impairment or dementia (odds ratio 1.5; 95% confidence interval 1.4–1.8).

Despite the current study’s strengths, which include a large sample size and well-controlled sociodemographic factors, there are several limitations. First, this is a cross-sectional study. Second, we did not have information such as the use of medication and treatment. Third, we only focused on a UK sample, and this may make it difficult to extrapolate the present findings to other cultures/countries. Future studies should test and analyze large-scale populations with high blood pressure in other cultures/countries. Fourth, cognition has more domains than the ones we assessed here. Thus, it is important for future studies to examine if other cognitive domains, such as working memory, are affected by high blood pressure. Finally, we did not have information regarding whether the participants are currently taking any medications that may have affected their cognition. Future studies should test the role of medication in relation to cognition in people with high blood pressure.

To conclude, the current study found that people with high blood pressure are characterized by impaired episodic memory, semantic verbal fluency, fluid reasoning, and numerical ability. This new study also has some implications. Clinicians and health professionals should consider including these tasks as part of the neuropsychological measurement for people with high blood pressure to detect their cognitive deficits. Moreover, they should also come up with ways to improve cognitive performance in people with high blood pressure.

## Figures and Tables

**Table 1:** Descriptive statistics of the demographic characteristics and cognitive performance of people with and without high blood pressure.

	People with High Blood Pressure		People without High Blood Pressure	
**Variable**	Mean	S.D.	Mean	S.D.
Age	59.71	15.39	59.37	17.02
Monthly income	1389.61	1253.29	1563.54	1651.06
Immediate task recall (standardized)	−0.24	0.99	−0.12	0.96
Delayed task recall (standardized)	−0.28	0.96	−0.13	0.97
Semantic verbal fluency (standardized)	−0.17	0.98	−0.01	1.00
Fluid reasoning (standardized)	−0.20	0.98	−0.07	0.98
Numerical ability (standardized)	−0.07	0.86	0.04	0.87
	N	%	N	%
**Sex**				
Male	1373	46.14	2358	46.09
Female	1603	53.86	2758	53.91
**Highest educational qualification**				
Below college	2256	75.81	3617	70.70
College	720	24.19	1499	29.30
**Legal marital status**				
Single	1226	41.20	1794	35.07
Married	1750	58.80	3322	64.93
**Residence**				
Urban	2215	74.43	3728	72.87
Rural	761	25.57	1388	22.96

**Table 2:** The ANOVA results from the model trained using the healthy controls for A. intermediate word recall, B. delayed word recall, C. animal naming, D. number series, and E. numeracy, respectively. SumSq = sum squared, DF = degrees of freedom, MeanSq = mean squared, F = F statistics, *p*-Value = *p*-value.

A. Immediate Word Recall					
Variable	SumSq	DF	MeanSq	F	*p*-Value
Age	335.95	1	335.95	436.34	*p* < 0.001
Sex	74.82	1	74.82	97.18	*p* < 0.001
Monthly income	12.09	1	12.09	15.70	*p* < 0.001
Highest educational qualification	142.77	1	142.77	185.44	*p* < 0.001
Legal marital status	6.90	1	6.90	8.96	*p* < 0.01
Residence	7.90	1	7.90	10.26	*p* < 0.01
Error	3933.5	5109	0.77		
**B. Delayed word recall**					
Variable	SumSq	DF	MeanSq	F	*p*-Value
Age	354.35	1	354.35	289.01	*p* < 0.001
Sex	78.26	1	78.26	8.36	*p* < 0.01
Monthly income	5.97	1	5.97	12.69	*p* < 0.001
Highest educational qualification	107.44	1	107.44	107.85	*p* < 0.001
Legal marital status	5.88	1	5.88	12.41	*p* < 0.001
Residence	9.28	1	9.28	25.15	*p* < 0.001
Error	4141.4	5109	0.81		
**C. Animal naming**					
Variable	SumSq	DF	MeanSq	F	*p*-Value
Age	258.5	1	198.86	233.84	*p* < 0.001
Sex	7.48	1	3.82	4.49	*p* < 0.05
Monthly income	11.35	1	11.28	13.26	*p* < 0.001
Highest educational qualification	96.47	1	96.47	107.85	*p* < 0.001
Legal marital status	11.10	1	13.21	15.54	*p* < 0.001
Residence	22.49	1	22.49	19.02	*p* < 0.001
Error	4569.7	5109	0.89		
**D. Number series**					
Variable	SumSq	DF	MeanSq	F	*p*-Value
Age	100.38	1	100.38	121.35	*p* < 0.001
Sex	42.75	1	42.75	51.68	*p* < 0.001
Monthly income	37.19	1	37.19	44.96	*p* < 0.001
Highest educational qualification	237.59	1	237.59	287.22	*p* < 0.001
Legal marital status	32.69	1	32.69	39.52	*p* < 0.001
Residence	13.26	1	13.26	16.03	*p* < 0.001
Error	4226.3	5109	0.83		
**E. Numeracy**					
Variable	SumSq	DF	MeanSq	F	*p*-Value
Age	15.37	1	15.37	23.68	*p* < 0.001
Sex	120.45	1	120.45	185.65	*p* < 0.001
Monthly income	28.72	1	28.72	44.27	*p* < 0.001
Highest educational qualification	185.66	1	185.66	286.15	*p* < 0.001
Legal marital status	29.30	1	29.30	45.16	*p* < 0.001
Residence	17.22	1	7.22	26.55	*p* < 0.001
Error	3314.8	5109	0.65		

## Data Availability

Data can be found at https://www.understandingsociety.ac.uk/documentation/mainstage (accessed on 1 March 2023).
